# Origin of the High-Frequency Shoulder in the Raman Spectra of CdSe Quantum Dots

**DOI:** 10.1021/acs.jpclett.4c02335

**Published:** 2024-10-09

**Authors:** Surender Kumar, Torben Steenbock, Gabriel Bester

**Affiliations:** † Department of Chemistry, University of Hamburg, HARBOR, Building 610, Luruper Chaussee 149, Hamburg 22761, Germany; ‡ Department of Chemistry and Physics, University of Hamburg, HARBOR, Building 610, Luruper Chaussee 149, Hamburg 22761, Germany; § The Hamburg Center for Ultrafast Imaging, Luruper Chaussee 149, Hamburg 22761, Germany

## Abstract

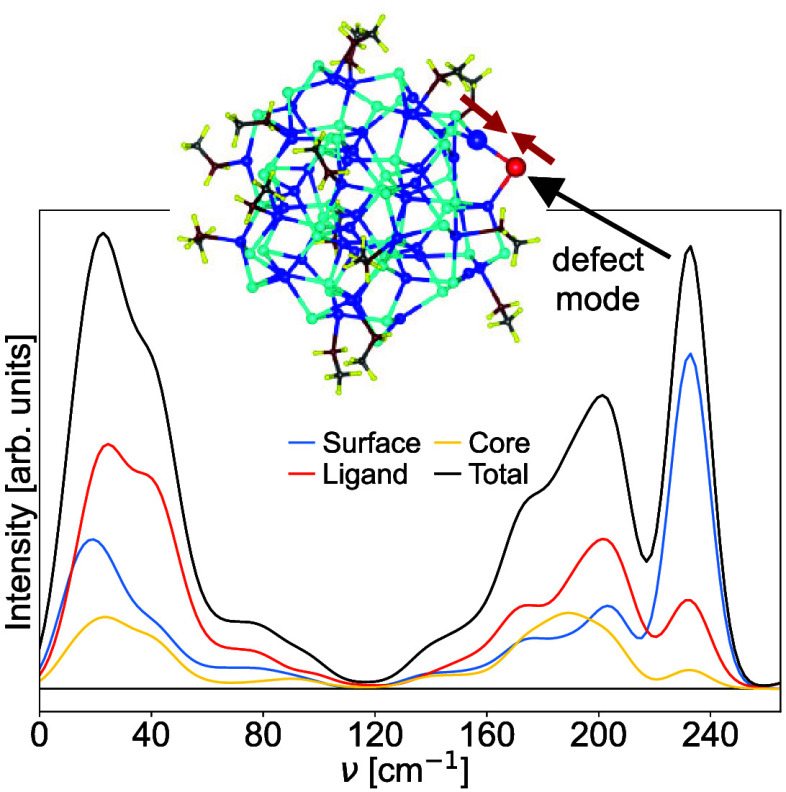

The origin of the high-frequency shoulder (HFS) observed above the longitudinal optical (LO) peak around 230 cm^–1^ in the Raman spectra of CdSe quantum dots (QDs) has been the subject of intense debate. We use state-of-the-art *ab initio* density functional theory applied to small CdSe QDs with various realistic surface passivations and find an intense Raman signal around 230 cm^–1^, which corresponds to a stretching vibration of a defective 2-fold coordinated Se atom. We interpret this signal as being the origin of the HFS. Since the signal disappears in fully passivated and defect-free (magic size cluster) structures, it can be used as a fingerprint to distinguish defective from nondefective structures.

Cadmium selenide (CdSe) quantum dots (QDs) from sub-2-nm (nm) to tens of nanometers^1−9^ have been extensively explored due to their size tunable optical properties. The latter are strongly influenced by vibrational properties which lead to the appearance of rich phonon sidebands^10−12^ and eventually shape the recombination dynamics.^13,14^ Raman spectroscopy is an effective technique to probe these vibrational properties,^15−24^ revealing, e.g., valuable information about alloying and strain at core/shell interfaces.^15,25,26^ For larger QDs (both core and core/shell), distinct longitudinal optical (LO) and surface-optical (SO) peaks have been observed in the Raman spectra. The behavior of these peaks, influenced by factors such as size, strain, and composition has been thoroughly characterized and understood.^16−18,25,27−31^ In addition to the well-established LO and SO peaks, Raman spectra of CdSe QDs often show a high-frequency shoulder (HFS) centered around 230 cm^–1^ above the LO region.^15−17,22,25,31^ The origin of this HFS remains debated, with hypotheses including amorphous surface selenium,^16^ surface bond reconstruction,^16^ and coupled optical-acoustic modes.^16^ The disappearance of the HFS upon CdS shell deposition^17,22,25^ suggests a surface-related origin, yet its precise nature is not fully understood.

We use density functional theory (DFT) to calculate the Raman intensity of realistic ligand-covered QDs with an emphasis on 2-fold coordinated Se-defect sites that have been shown to play an important role in small CdSe QDs.^32−34^ While simplified models accurately predict Raman spectra of larger structures,^18,27,35,36^ they are inadequate for capturing the surface effects that will turn out to be crucial for smaller QDs. Prior *ab initio* Raman studies focused on defect-free structures^24,37,38^ leaving the importance of defects mainly unexplored from a theoretical standpoint.

We show that the 2-fold coordinated surface Sedefect, where the Se atom is bonded to a 3-fold coordinated Cd on one side and to a 4-fold coordinated Cd on the other side, and hence an asymmetric bonding situation, leads to a very strong Raman signal at the frequency corresponding to the experimentally observed HFS frequency. Fully passivated surfaces and core/shell structures do not show any significant peak in this frequency region, making the observation of the HFS a clear signal of the presence of these types of Sedefects. Furthermore, we show that a realistic surface termination, including L-type ligands, leads to a much enhanced Raman activity in the optical region in general, compared to structure with an idealized passivation or core/shell structures.

In Figure 1, we show the relaxed wurtzite nanoclusters and QDs investigated in this work. The smallest structures (also experimentally realized), Cd_13_Se_13_^39^ and Cd_33_Se_33_,^24,39,40^ are called magic-size clusters and have 3-fold coordinated Se that do not develop a defect state in the gap^5,32−34,41^ and 3-fold coordinated Cd atoms that are partly passivated by methylamine (MA; with a shortened alkyl chain compared to the experiment). This common L-type ligand selectively binds to surface Cd atoms^2−4,42^ via a covalent dative bond, where the nitrogen atom in MA donates an electron pair to the Cd atom. Some of the surface Cd that remains unpassivated and 3-fold coordinated does not form defect states in the gap. In this sense, magic-size clusters are free from defects. We keep the stoichiometry at 50% and increase the size to Cd_45_Se_45_. For this nonmagic-size QD, three 2-fold coordinated Se atoms (defects) are unavoidable in the QD construction. The top right panel of Figure 1 gives a detailed view of the defect geometry: Se is bonded to a 3-fold Cd on one side and to a 4-fold Cd on the other side. We supplement the portfolio of calculations by a trioctylphosphine oxide (TOPO, where octyl chains are replaced by methyl groups to reduce computational cost) passivated structure as well as pseudohydrogen (PH)^43−45^ passivated structures. These pseudohydrogen atoms have a fractional charge of 3/2 (1/2) when attached to Cd (Se) and lead to electronic gaps free of defect states.^27,35^ We can see these structures as “ideally” passivated. The L-type ligand coverage in our TOPO and MA structures is different; following the literature^46,47^ we use a larger coverage for the smaller MA molecule (15) than for the bulky TOPO (12). The Cd_45_Se_45_/Cd_123_S_129_–PH structure is a core/shell structure where the CdSe core has the same size as the Cd_45_Se_45_–PH QD. Computational limitations prohibit the use of larger structures.

**Figure 1:**
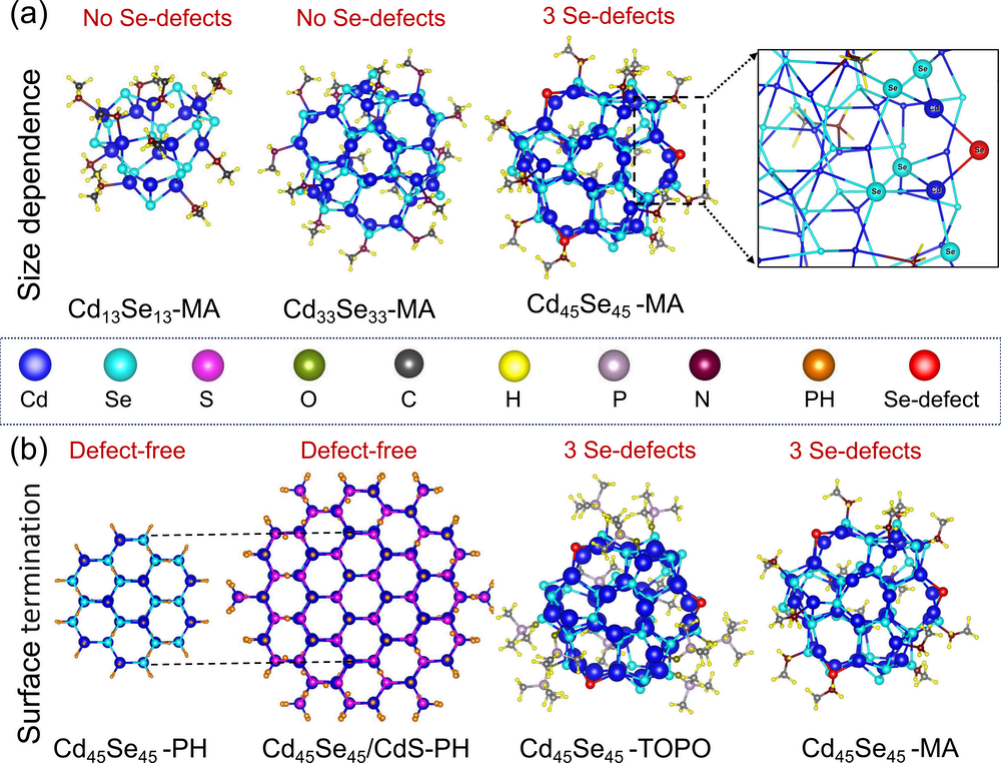
Optimized structure of (a) methylamine (MA) passivated ultrasmall CdSe nanoclusters and QDs of varying sizes, represented by the number of constituent atoms. (b) Different surface termination of the Cd_45_Se_45_ structure including pseudohydrogen (PH), a CdS shell with PH on top, and trioctylphosphine oxide (TOPO). The inset provides an enlarged view of the local arrangement around the 2-fold coordinated Sedefect, highlighted in red. The dotted lines delineate the core size in the core/shell structure.

In Figure 2, we show the single particle eigenvalues (a), isosurfaces of the defect molecular orbitals (DMO; b) of the highest occupied molecular orbital (HOMO), and the lowest unoccupied molecular orbital (LUMO; c). The isosurface values are chosen in such a way as to enclose 75% of the state density.

**Figure 2:**
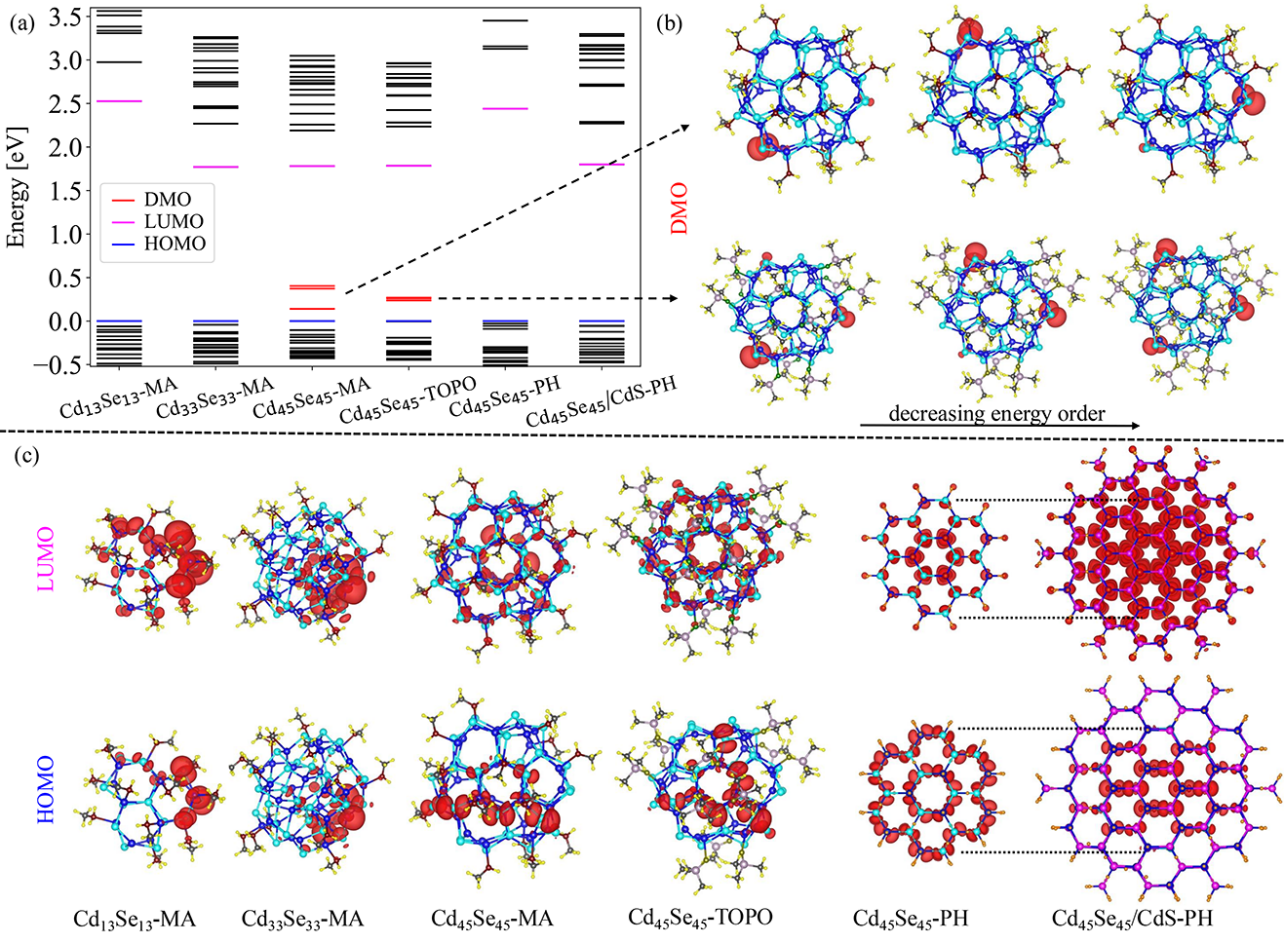
Electronic structure analysis. (a) Single-particle energy levels for the various structures (see Figure 1). The HOMO, LUMO, and DMO are shown as blue, magenta, and red lines, respectively. The HOMO level is set to 0 eV for all structures. Isosurfaces of the wave function squared enclosing 75% of the state density for the DMOs (b), LUMO, and HOMO (c).

In Figure 2a, we see that only the structures declared as defective (Cd_45_Se_45_-MA and Cd_45_Se_45_-TOPO) show defect levels (doubly occupied, shown in red) within the HOMO–LUMO gap. For these states, the wave function is strongly localized on the Sedefect (Figure 2b). For the Cd_45_Se_45_-MA structure, the three defect levels are energetically split by as much as 263 meV, while for Cd_45_Se_45_-TOPO they remain nearly degenerate, so that the individual DMO look artificially distributed over the three defect sites. We understand the splitting in Cd_45_Se_45_-MA as follows. In Cd_45_Se_45_-TOPO, the L-type ligands are placed in a relatively symmetric arrangement (although the structure has strictly speaking no remaining symmetry), and the three defect sites are nearly equivalent. The three additional L-type ligands in Cd_45_Se_45_-MA break the symmetry (further), rendering the three defects inequivalent. A comparison of both structures (MA and TOPO) is given in the Supporting Information (SI). It is also interesting to see that the HOMO–LUMO gaps of the smaller Cd_33_Se_33_ and the larger Cd_45_Se_45_ structures are nearly the same. This is a consequence of the hybridization of the DMO with the HOMO which pushes the HOMO to lower energy, opening the HOMO–LUMO gap in the defective larger structure.

In Figure 2c, we notice that the HOMO tends to be more localized than the LUMO, in accordance with the heavier hole effective mass. This is also true for the magic size clusters we described earlier as “defect-free” and now exhibit a hole localization in an off-center location. It is clear that a static calculation for a chosen geometry, such as the one performed here, is only a snapshot of the true very dynamic situation where L-type ligands wiggle and presumably even interchange attachment sites.^48^ The PH-passivated structures show almost no geometric relaxation, and the HOMO and LUMO states are accordingly highly symmetric. The core/shell Cd_45_Se_45_/CdS-PH structure shows a quasi type-II character, where the hole is localized in the core and the electron is over the entire structure.

The calculated Raman spectra are shown in Figure 3, for the Cd_13_Se_13_-MA, Cd_33_Se_33_-MA, and Cd_45_Se_45_-MA structures. The top panels (a–c) are plotted using a Gaussian broadening of 23.5 cm^–1^, while the bottom panels (d–f) show unbroadened results. We use two types of analysis of the signal; on the top, we discriminate surface/ligand/core contributions according to eq 1, and on the bottom we use the inverse participation ratio (IPR)^49^ from eq 2. A low value of IPR (red) signifies localized vibrations. For the lower panels, we normalized the intensities according to the breathing mode, which is easy to identify. While this specific normalization is debatable, it is at least rather simple to comprehend. We focus on the region below 250 cm^–1^, so that intraligand vibrations, typically up to around 3000 cm^–1^,^38^ are not presented.

**Figure 3:**
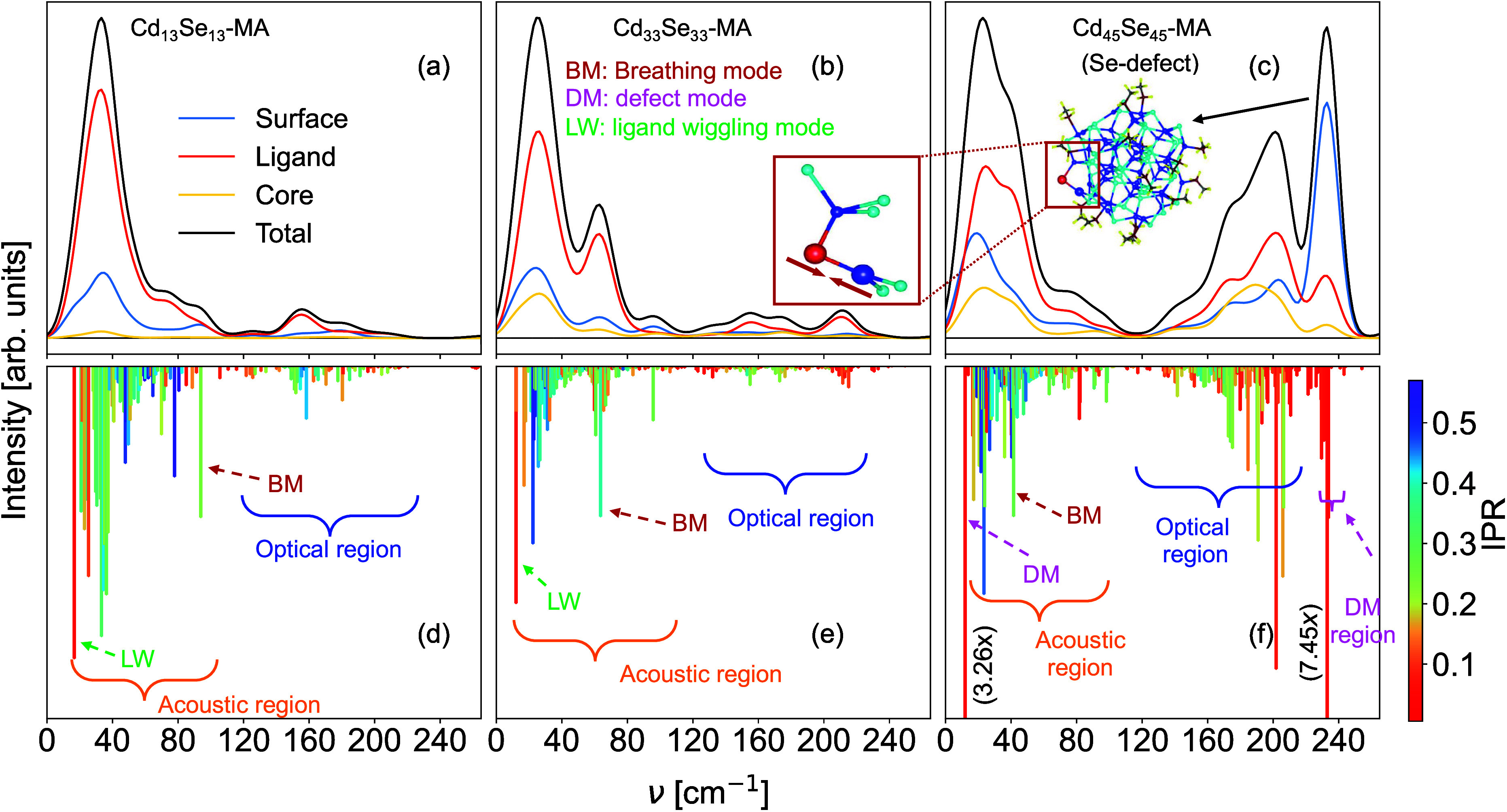
Calculated Raman spectra of (a,d) Cd_13_Se_13_-MA, (b,e) Cd_33_Se_33_-MA, and (c,f) Cd_45_Se_45_-MA using a Gaussian broadening of 23.5 cm^–1^ (solid lines upper panels, a–c) and without broadening (bars on the lower panels, d–f). The broadened results are analyzed in terms of the three fragments: ligand, surface, and core (eq 1). The bars (bottom panels) are colored according to the IPR value (eq 2). The inset in c shows the QD highlighting the defect, and the inset in b shows the vibrational eigenmode of the defect vibration at 233 cm^–1^.

As is common for semiconductors, the vibrations can be divided into an acoustic and an optical region, as indicated in the lower panels of Figure 3. We notice that for the smaller magic size clusters the optical region is nearly Raman-inactive, so that dominant contributions originate from acoustic type modes. The optical region has become active already for the slightly larger Cd_45_Se_45_-MA structure. It is known that the LO peak strongly dominates for larger, bulk-like structures,^28−30^ and the emergence of the LO peak with QD size was investigated earlier.^23^ We see in Figure 3c that the rise of the optical region correlates with the emergence of modes with significant core characters (yellow line). Generally, we see that many vibrations are to a certain extent Raman active, which makes a meaningful and concise analysis difficult. However, we can identify a few distinctive modes. The breathing modes (BM in Figure 3d–f, see SI for movies) show the qualitative frequency shift we would expect from the Lamb model,^35^ i.e., to the red when the size increases: 93.86 cm^–1^, 63.66 and 41.53 cm^–1^ for Cd_13_Se_13_-MA, Cd_33_Se_33_-MA, Cd_45_Se_45_-MA, respectively. One further mode that strikes by its low IPR value (strong localization) and strong intensity is labeled as LW in Figure 3d–f). It corresponds to a wiggling motion of the entire ligand molecule (MA). This vibration will most likely be hindered in a realistic setup including solvents, and we highlight it here to point at possible pitfalls in the raw computational results. Our most interesting result is the appearance of very intense defect-related peaks in the low (11.93 cm^–1^) and the high-frequency regions (229.5, 231.1, and 232.99 cm^–1^) of Cd_45_Se_45_-MA spectra (marked DM in Figure 3f). Each of the three Sedefects produces one high-frequency Raman peak, while we could identify only one low-frequency defect-related peak. This low-frequency mode corresponds to the wagging vibration of the 2-fold coordinated Sedefect, where the Se moves perpendicular to the Cd–Se–Cd plane. The nature of the high-frequency mode is shown in the inset of Figure 3b. The Se and the 3-fold coordinated Cd undergo a stretching vibration, which is known to be strongly Raman active. The other Se–Cd bond (toward the 4-fold Cd) accommodates the stretching but with very small bond-length change.

Note that the 3-fold coordinated Cd atoms, present at the surface of all our structures, reconstruct into a planar geometry with high symmetry. The localized vibrations in these regions exhibit a symmetric Raman-inactive character.

As a next step, we will investigate the influence of the surface on the Raman intensity and show in Figure 4 results for the same size QD, but with different terminations. Figure 4d,h) is a repetition of Figure 3c,f and is used here again for ease of comparison. The simplest structure is Cd_45_Se_45_–PH which has idealized passivation and high symmetry. As expected, none of the defect features previously mentioned are present. The breathing mode at 55 cm^–1^ is the most intense, with a high IPR value, indicating that many atoms vibrate collectively, as expected from a breathing mode in high symmetry. These types of modes are strongly Raman active. We also notice only very weak intensities in the optical region and it seems that lower symmetry (realistic surfaces) leads to an activation of the optical region already at smaller sizes. Covering the QD with a shell of CdS leads to the Cd_45_Se_45_/CdS-PH core/shell structure shown in Figure 1b. The breathing mode still dominates at a somewhat lower frequency of 41.8 cm^–1^, in accordance with the larger size of the structure. The LO peak starts to appear at 194 cm^–1^.

**Figure 4:**
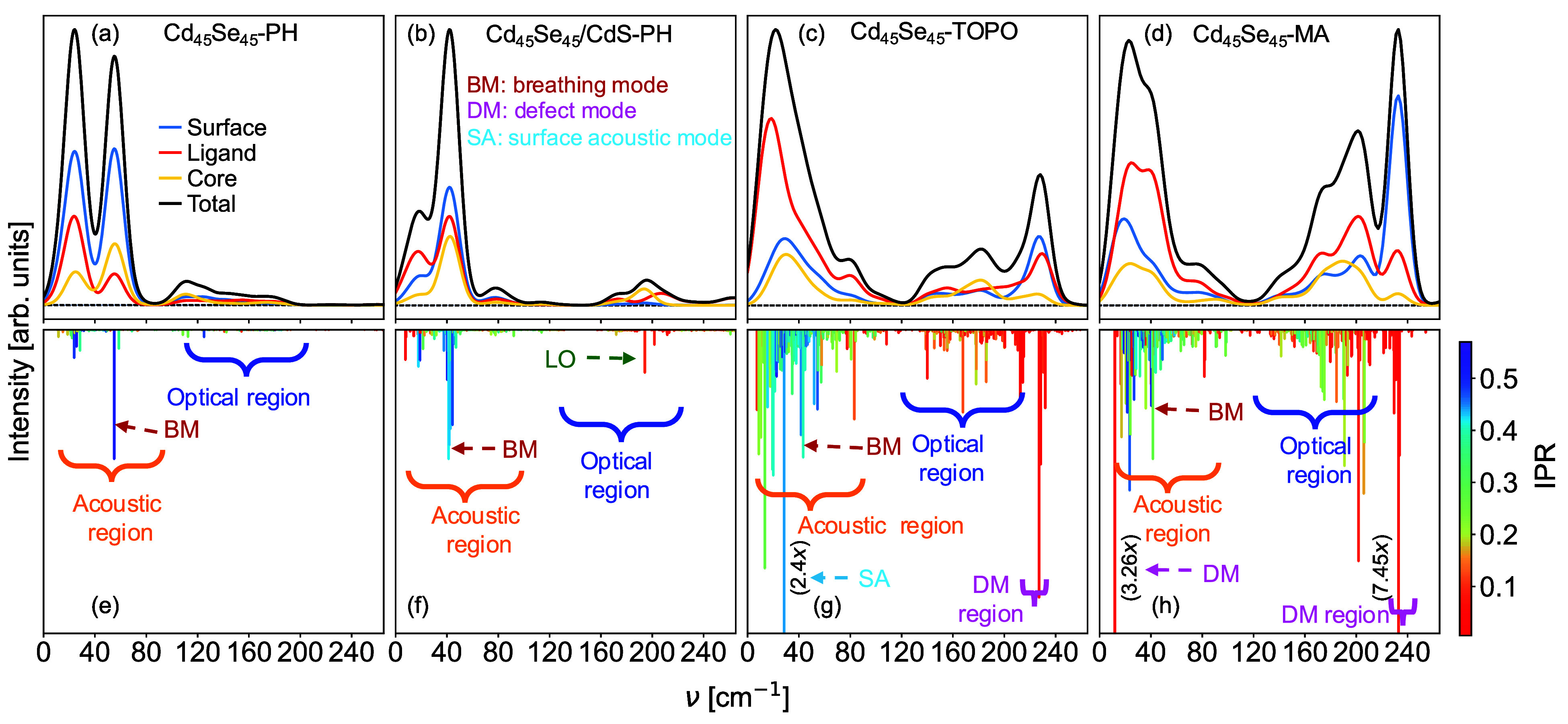
Similar to Figure 3 but depicting Cd_45_Se_45_–PH (a,e), Cd_45_Se_45_/CdS-PH (b,f), Cd_45_Se_45_-TOPO (c,g), and Cd_45_Se_45_-MA (d,h).

While both the idealized passivated and the core/shell structures lead to the absence of defect peaks, the TOPO covered structure shown in Figure 4c,g resembles one of the MA passivated structure. The breathing mode for TOPO and MA terminated structures has similar frequencies (43.39 and 41.53 cm^–1^, respectively) and has in both cases significant intensities. The optical region is somewhat more intense in the case of the MA terminated structure. Although the L-type ligands bind only very weakly to the surface Cd atoms, they have an influence on the Raman activity. In the case of TOPO, oxygen binds to Cd, while it is nitrogen for MA. Also, the structure is affected by the type of ligand, although to a relatively low extent (both relaxed atomic positions are compared in SI). For Cd_45_Se_45_-TOPO, we observe the high-frequency defect peaks that we described for Cd_45_Se_45_-MA but do not observe the low-frequency DM peak, which hints at a strong surface dependence of this specific vibration. We find, however, a vibration of acoustic character that is rather delocalized over a large part of the surface (on one “facet”) and indicated it with SA, for surface acoustic^35^ in Figure 4g. One further piece of information can be extracted from the calculations with respect to the defect peaks. The vibrational frequencies of the three peaks corresponding to the three defects are slightly split—227.1 cm^–1^, 227.2 cm^–1^, and 228.5 cm^–1^ for TOPO and 229.5 cm^–1^, 231.1 cm^–1^, and 232.99 cm^–1^ for MA—and have significantly different intensities. We notice that the splitting in vibrational frequencies corresponds to the splitting in the DMO energies in the sense that we obtain large splittings in eigenvalues (263 meV) and frequencies (3.49 cm^–1^) for MA and small splittings in eigenvalues (32 meV) and frequencies for (1.4 cm^–1^) TOPO. We described this earlier as symmetry inequivalent Sedefects. If we look at the bond length on one and the other side of the 2-fold coordinated Sedefect (see SI), we notice that the strongest Raman intensity occurs for the situation where the imbalance is the greatest. In other words, the Raman intensities seem to be correlated to the difference in the Se–Cd bond lengths. Our largest difference is 0.039 Å (2.517 vs 2.556 Å) for the defect with the largest Raman intensity at 232.99 cm^–1^ in Cd_45_Se_45_-MA.

To rule out the possibility that amorphous Se is responsible for the HFS, as previously suggested,^16^ we perform calculations on a Cd_13_Se_13_-MA cluster with an additional neutral Se atom. The introduction of the Se atom leads to notable bond reconstruction, forming a Se–Se dimer (see SI), and no states within the gap, but rather above the LUMO.^34^ Raman spectra reveal that both Cd_13_Se_13_-MA and Cd_13_Se_14_-MA clusters exhibit similar features without Raman-active HFS defect vibrations near 230 cm^–1^ (see SI), unlike the Cd_45_Se_45_ structure.

We finally compare our results with the experimentally measured spectra reported by Moses Badlyan et al.^17^ and reproduced in Figure 5. The authors reported a high-frequency shoulder (HFS), shown in magenta, above the optical region around 233 cm^–1^ in pure CdSe QDs, which is shown to disappear upon growth of a CdS shell. This makes their data particularly relevant for direct comparison with our results. A similar HFS has been observed in QDs of different sizes, ranging from 1.44 to 3.4 nm.^15,16,22,25,31^ Our calculated defect peaks, located above the optical region (228 cm^–1^ for TOPO and 233 cm^–1^ for MA), coincide with the experimentally observed HFS. Additionally, our calculations for the core/shell structure, as presented in Figure 4b, do not exhibit this peak, which is consistent with the experimental findings. However, in the experimental data, the high-frequency shoulder (HFS) is relatively weak compared to the signals from the optical region, which is in contrast to our calculations. We attribute this discrepancy to two main factors. First, the size differences between the QDs in the experiments and our calculations certainly play a significant role, especially in the intensity of the LO peak. Our QD sizes only allow for a partial observation of the LO peak that is known to become very dominant as the size increases. Second, it is most likely that not all of the QDs in the experimental sample are defective, which would naturally lead to a weaker defect peak.

**Figure 5:**
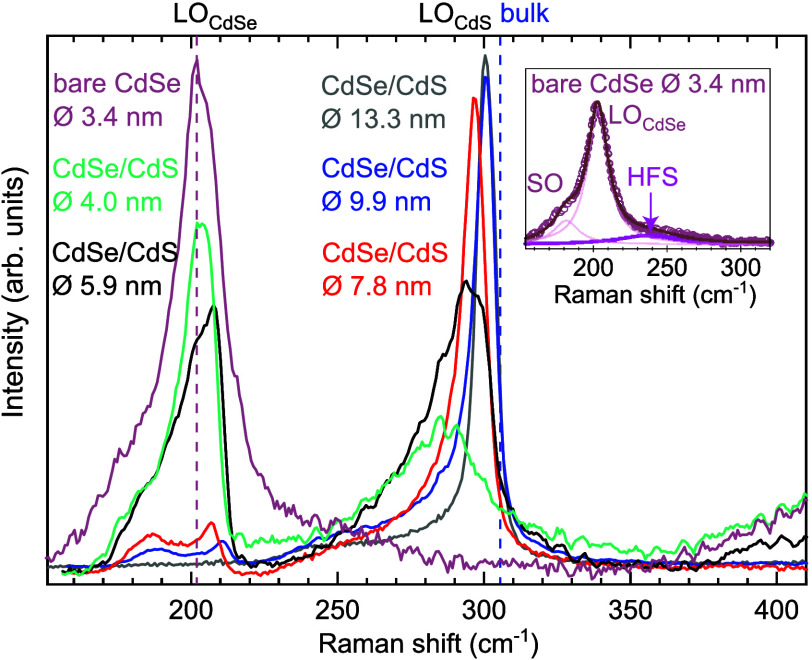
Measured Raman spectra of bare CdSe core with a diameter (ϕ) 3.4 nm and CdSe/CdS core/shell QDs with different shell thicknesses. Reproduced with permission from ref (17). Copyright 2019 American Physical Society. Inset: Spectrum of the bare CdSe QD, highlighting the HFS in magenta. Similar HFS has been measured in other experiments.^15,16,22,25,31^

The fact that similar HFSs have been reported for other II–VI QDs (CdS, CdTe, ZnTe)^16,31,50^ leads to the natural question to whether our defect related HFS could explain these results as well. While we were not able to perform further calculations within this work, the defect vibration can be expected to be present in other II–VI QDs. Indeed, 2-fold coordinated S or Te atoms (if present in the structure) could undergo the asymmetric stretching mode described here. In terms of frequency, we would expect to see it on the blue side of the LO peak as well since the defect vibration has similarities with an LO vibration but with a more loosely bound chalcogen atom. However, this remains speculative at the moment.

In summary, we identify, via state-of-the-art *ab initio* DFT on small CdSe QDs with various realistic surface passivations, an intense Raman signal at approximately 230 cm^–1^, corresponding precisely to the frequency at which the debated HFS is experimentally measured. This signal corresponds to the stretching vibration of a defective 2-fold coordinated Se atom. The Se atom is bonded to two inequivalent Cd atoms, one being 3-fold and the other 4-fold coordinated. We observe that the inequivalence of the two bonds leads to the asymmetric geometry and to the very intense Raman signal. The placement and the type of L-type ligands (TOPO or MA) influence this inequivalence and hence the intensity of the signal. Nevertheless, the signal remains significantly intense in all scenarios. To compare with experimental observations, which reported the disappearance of the HFS after the growth of a protective shell, we constructed a core/shell structure and observed the same phenomenon. Accordingly, the signal disappears in defect-free (magic size cluster) structures, making it a distinctive marker for identifying defective versus nondefective structures. We further emphasize that introducing a neutral amorphous Se atom does not result in any Raman-active vibrations in the region of the HFS. More generally, we observe that the Raman signal in the optical vibrational region is activated when realistic, partly reconstructed, L-type ligand-protected surfaces are considered. In contrast, QDs modeled with idealized passivation, and thus higher symmetry, exhibit comparatively weaker Raman signals in this spectral region.

## Method

All calculations were carried out using the TurboMole 7.5 program package^51^ utilizing the Perdew–Becke–Enzerhoff (PBE) exchange-correlation functional,^52^ Ahlrich’s double-ζ split-valence basis set with polarization functions on all atoms (def2-SVP), and Grimme’s empirical dispersion correction in the third generation (D3).^53^ All structures are optimized with a convergence of 10^–7^ Hartree for energy and 10^–6^ Hartree/Bohr for the gradients. Raman scattering cross-sections at incident wavelength 850 nm are calculated from the derivative of the polarizability (see SI). Calculations using a larger triple-ζ def2-TZVP basis set yield very similar results (see SI), justifying the use of the def2-SVP basis set. We calculate the contributions to the total Raman spectra of QDs from three distinct fragments: ligands, the bulk-like core (Cd 4-fold coordinated with Se and vice versa), and the surface (Cd atoms not 4-fold coordinated with Se and vice versa). To understand how fragments of the QD contribute to the Raman spectra of CdSe QDs, we calculate the contributions from specific fragments of the structure to the *Q*_p_ vibrational mode using
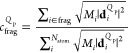
1where *i* is atom indices, *M*_*i*_ is the mass of atom *i*, and **d**_*i*_^*Q*_p_^ is the normalized atomic displacement vector. *c*_frag_^*Q*_p_^ ranges between 0 (no contribution) and 1 (100% contribution) to the vibrational mode.

The degree of localization for vibrational modes is analyzed using the inverse participation ratio (IPR):^49^
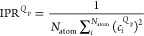
2where *c*_*i*_^*Q*_p_^ represents the contribution of atom *i* to the *Q*_p_th vibrational mode. The IPR ranges from 1 (if all atoms contribute) to 1/*N*_atom_ (if only a single atom contributes).

## Supporting Information Available

Theoretical description of Raman intensity calculations; the impact of basis set size on Raman spectra; comparison of the relaxed geometries and Se-defect bond lengths of Cd_45_Se_45_ with MA and TOPO ligands; Raman spectra with amorphous Se atom on the surface; Raman spectra of Cd_45_Se_45_/CdS-PH core/shell structures (highlighting the optical region of the CdS shell omitted in the main text) (PDF)
Cartesian coordinate (xyz) files for all structures under investigation (ZIP)
Movies of different vibrational modes (ZIP)
Transparent Peer Review report available (PDF)
