# Supplementary material for: Origin of the High-Frequency Shoulder in the Raman Spectra of CdSe Quantum Dots
Source: J Phys Chem Lett. 2024 Oct 9;15(41):10392–8. doi: 10.1021/acs.jpclett.4c02335 (PMC11492379; doi:10.1021/acs.jpclett.4c02335)
Supplement: Supplementary file 1 — jz4c02335_si_001.pdf [file jz4c02335_si_001.pdf]

Supporting Information: Origin of the High-Frequency Shoulder in the Raman Spectra of CdSe Quantum Dots

Surender Kumar,[†] Torben Steenbock,[†] and Gabriel Bester[*,‡,¶]

†*Department of Chemistry, University of Hamburg, HARBOR, Building 610, Luruper Chaussee 149, Hamburg, 22761 Germany.*

‡*Department of Chemistry and Physics, University of Hamburg, HARBOR, Building 610, Luruper Chaussee 149, Hamburg, 22761 Germany.*

¶*The Hamburg Centre for Ultrafast Imaging, Luruper Chaussee 149, Hamburg, 22761 Germany.*

E-mail: gabriel.bester@uni-hamburg.de

Theory of Raman calculations

The vibration (or phonon) calculations are based on the solution of the dynamical equation:[1–3]

$$\sum_{I\alpha} D_{I\alpha,J\alpha'}\, X^{p}_{I\alpha} \;=\; \omega_p^2\, X^{p}_{J\alpha'} \;, \tag{S1}$$

where $I, J = 1, \ldots, N_{at}$ (with N_{at} the number of atoms) are atomic indices, α, α' are their corresponding Cartesian coordinate indices, p is the vibrationic/phononic index, and X^p are the normal modes of vibration. The dynamical matrix D is defined as

$$D_{I\alpha,J\alpha'} \;=\; \frac{1}{\sqrt{M_I M_J}}\, \frac{\partial^2 \mathcal{E}^{BO}}{\partial R_{I\alpha}\partial R_{J\alpha'}} \;, \tag{S2}$$

where $M_{I,J}$ are the atomic masses, $R_{I,J\alpha}$ are the nuclear coordinates and $\mathcal{E}^{BO}$ is the Born-Oppenheimer energy surface;[1,2] ω_p^2 and X^p are the eigenvalues and eigenvectors of the dynamical matrix (ω_p are the vibration/phonon frequencies).

Once the vibrational eigenvalues ω_p and eigenvectors X^p are obtained, Raman scattering cross sections can be calculated using the derivatives of the frequency-dependent polarizability tensor w.r.t. normal vibrational modes. The Raman scattering differential cross-section ($\frac{d\sigma}{d\Omega}$) in terms of frequency-dependent polarizability tensor is given as:[4,5]

$$\left(\frac{d\sigma}{d\Omega}\right)_p = k_\omega \left[45\left(\frac{d\alpha}{dQ_p}\right)^2 + 7\left(\frac{d\gamma}{dQ_p}\right)^2\right], \tag{S3}$$

where $\alpha'(\omega)$ and $\gamma'(\omega)$ are the isotropic part and the anisotropic part of the differentiated polarizability tensor, respectively at incident frequency ω. k_ω is a pre-factor given as:

$$k_\omega = \frac{\hbar}{4\,\pi\,\varepsilon_0^2\,c^4} n_v \frac{(\omega-\omega_p)^4\,g_p}{2\,\omega_p}, \tag{S4}$$

where c is the speed of light, frequency of vibration (ω_p) (or the phonon) and degeneracy of vibration (g_p) and ε_0 stands for the dielectric constant of vacuum. n_v is the occupation of vibrational levels at temperature T and included through Boltzmann distribution $1/(1-e^{h\,c\,\omega_p/k_B T})$ with h and k_B being Planck and Boltzmann constants respectively. The isotropic part α is given as:

$$\alpha = \frac{1}{3}\left(\alpha_{xx}+\alpha_{yy}+\alpha_{zz}\right), \tag{S5}$$

and the anisotropic part γ^2 is given as:

$$\begin{aligned}\gamma^2 = \tfrac{1}{2}\left[(\alpha_{xx}-\alpha_{yy})^2+(\alpha_{yy}-\alpha_{zz})^2+(\alpha_{xx}-\alpha_{zz})^2\right]+\\ +\tfrac{3}{4}\left[(\alpha_{xy}+\alpha_{yx})^2+(\alpha_{xz}+\alpha_{zx})^2+(\alpha_{yz}+\alpha_{zy})^2\right].\end{aligned} \tag{S6}$$

The polarizability tensor α is calculated from the analytical derivatives of a fully variational polarizability Lagrangian as implemented in the Turbomole package.[5]

Influence of basis set size on the Raman spectra

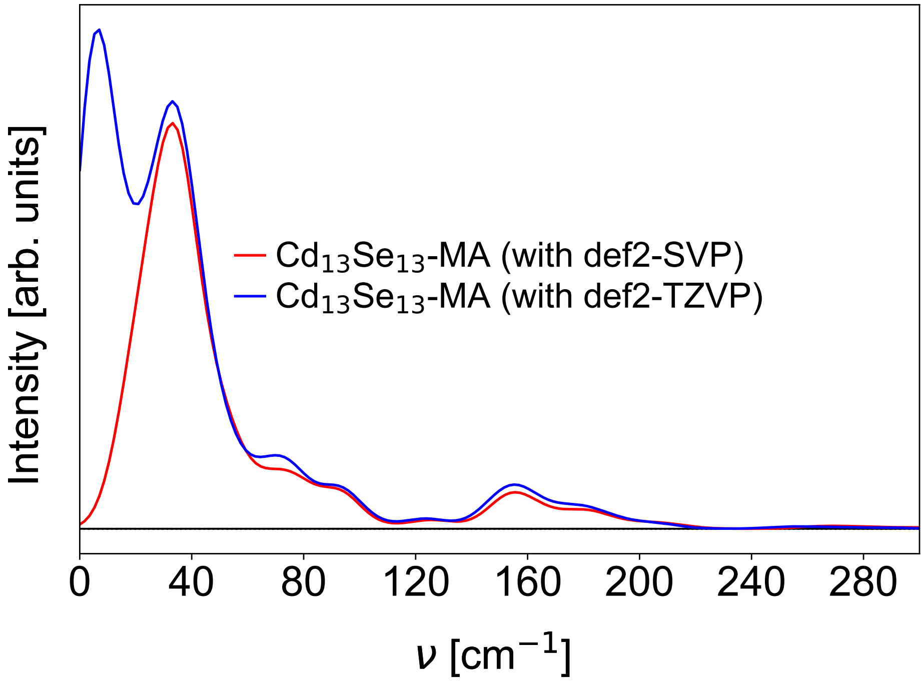

Figure S1: Comparison of the calculated Raman spectra for the $Cd_{13}Se_{13}$-MA structure using a smaller double-ζ def2-SVP (red) basis set and a larger triple-ζ def2-TZVP (blue) basis set.

In Fig. S1, we confirm that the Raman spectra remain consistent despite using larger basis sets by comparing spectra obtained with the smaller double-ζ def2-SVP and the larger triple- ζ def2-TZVP basis sets for the $Cd_{13}Se_{13}$-MA structure. Both calculations have been performed with the PBE exchange-correlation functional. The results indicate that the Raman spectra do not change in the higher-frequency regions with larger basis sets. The only noticeable change is in the lowest frequency ligand wiggling mode (LW mode in the main text), which shifts to a lower frequency with the larger def2-TZVP basis set. As discussed in the main text, the LW mode is expected to be suppressed in solution due to the dynamic nature of ligands. This finding justifies the use of the computationally cost-effective double-ζ def2-SVP basis set for the calculations presented in the main text. A similar observation was reported by Manav and co-workers,[6] who noted negligible differences in the Raman spectra of CdSe structures when using different basis sets.

$Cd_{45}Se_{45}$-MA and $Cd_{45}Se_{45}$-TOPO structure comparison

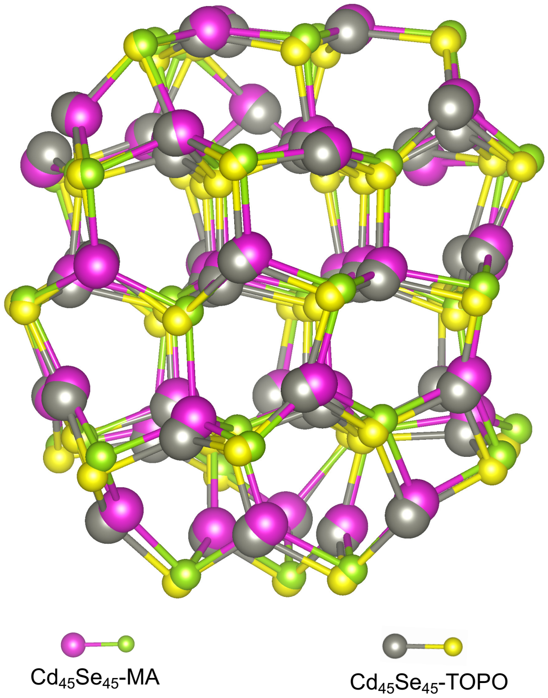

Figure S2: Comparison of optimized structure of $Cd_{45}Se_{45}$ with MA and TOPO passivants. Ligands are not shown here.

Table S1: Bond length (in Å) of three Se-defects in $Cd_{45}Se_{45}$-MA and $Cd_{45}Se_{45}$-TOPO structures.

Coordination	3-fold Cd	4-fold Cd
MA	2.517	2.547
	2.517	2.556
	2.542	2.565
TOPO	2.528	2.544
	2.528	2.544
	2.529	2.543

In Table S1, we present the bond lengths for all three Se-defects attached to a threefold-coordinated Cd on one side and a fourfold-coordinated Cd on the other, for both $Cd_{45}Se_{45}$-MA and $Cd_{45}Se_{45}$-TOPO structures.

Raman spectra with amorphous Se atom on the surface

Given that amorphous Se surface atoms have been proposed as a potential source of the high-frequency shoulder (HFS),[7] we examined this scenario by introducing a neutral Se (0) atom, representing amorphous Se, into the $Cd_{13}Se_{13}$-MA magic cluster. As discussed in the main

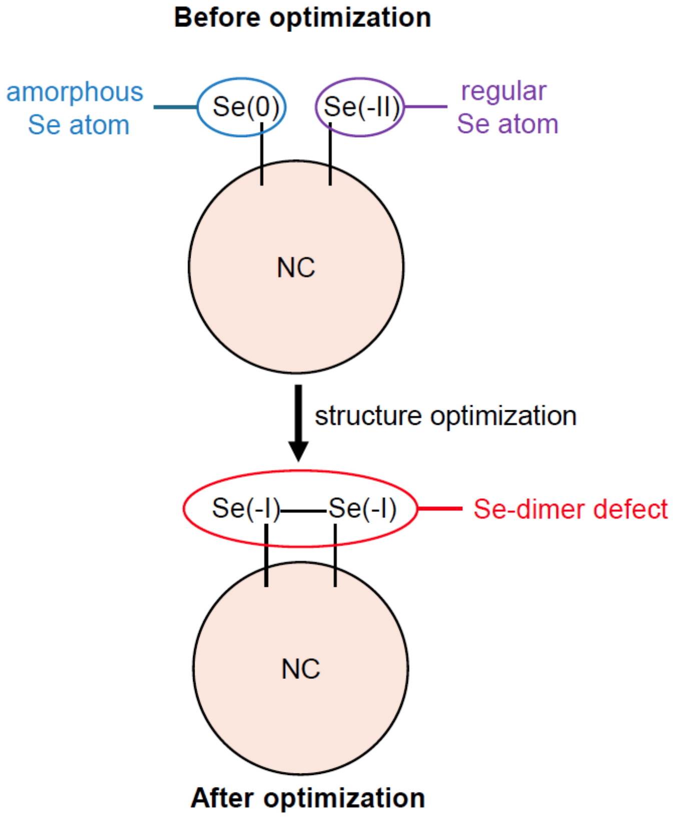

Figure S3: Schematic of Se-Se dimer formation in $Cd_{13}Se_{14}$-MA with neutral Se (0) surface atom.

text, this cluster does not show any Raman-active vibrations in the HFS region, making it an ideal test case for assessing whether the amorphous Se (0) atom could induce Raman-active vibrations in the HFS region, similar to those observed in $Cd_{45}Se_{45}$-MA/TOPO structures. After geometry optimization, we observed that the neutral Se (0) atom prompts significant bond reconstruction, which is depicted in Fig. S3. The newly added Se (0) atom forms a Se (-I)-Se (-I) dimer with an existing Se (-II) atom on the surface, where Se (-II) is oxidized and Se (0) is reduced.

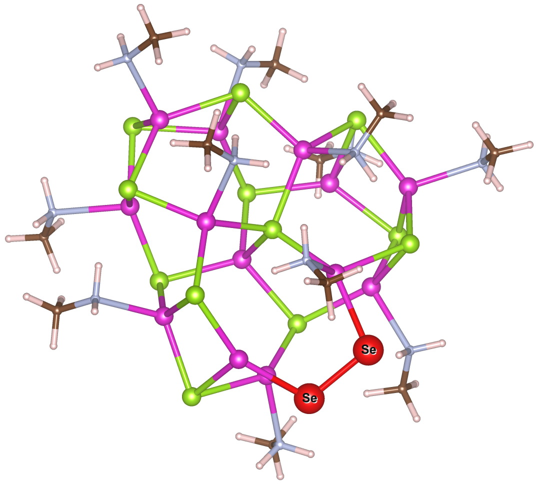

Figure S4: The optimized geometry of $Cd_{13}Se_{14}$-MA resulted from the addition of a neutral Se atom to $Cd_{13}Se_{13}$-MA nanocluster (NC) structure. The resulting structure features a Se-Se dimer on the surface, highlighted in red.

The optimized geometry of $Cd_{13}Se_{14}$-MA is shown in Fig. S4, with Se-Se dimer highlighted in red. The formation of Se-Se dimers has been reported in the literature and it is well known that these dimers do not introduce defect states into the band gap.[8]

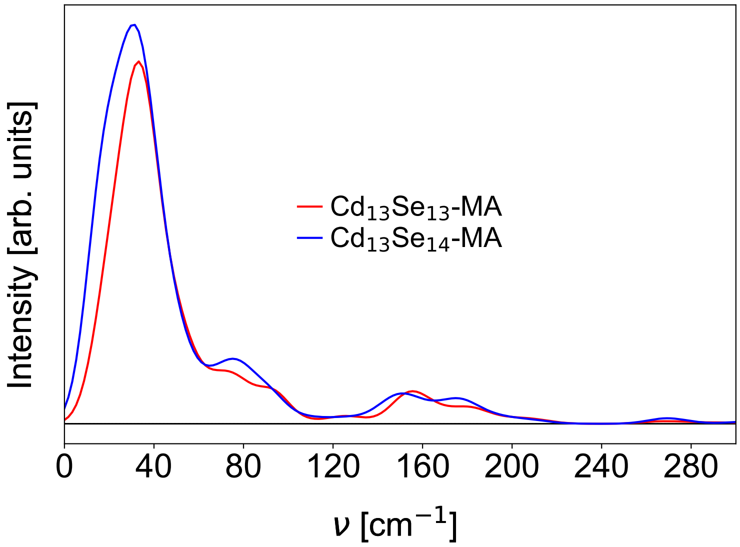

Figure S5: Comparison of the calculated Raman spectra for the $Cd_{13}Se_{13}$-MA (red line) and $Cd_{13}Se_{14}$-MA (with neutral Se surface atom) (blue line) structures.

In Fig. S5, we show the comparison of calculated Raman spectra of $Cd_{13}Se_{13}$-MA (red) and $Cd_{13}Se_{14}$-MA (with an extra Se atom) (blue) structures. Both the spectra display similar characteristics and lack any Raman-active vibrations in the region around 230 cm^{-1}, in contrast to the $Cd_{45}Se_{45}$-MA/TOPO structures. These results potentially provide results that amorphous Se surface atoms might not be the origin of HFS.

$Cd_{45}Se_{45}\backslash$CdS-PH core-shell spectra

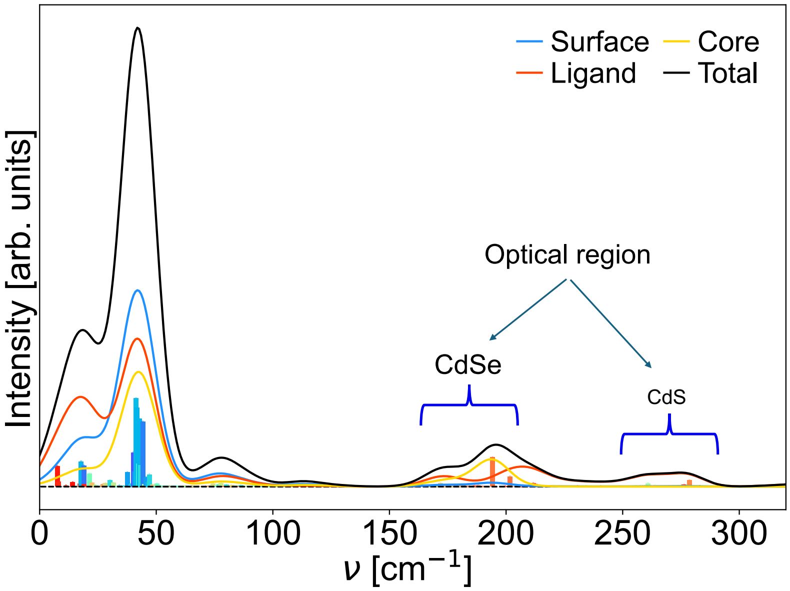

Figure S6: Calculated Raman spectra of $Cd_{45}Se_{45}\backslash$CdS-PH core-shell showing the optical regions for both CdSe core and CdS shell.

References

(1) García-Risueño, P.; Han, P.; Kumar, S.; Bester, G. Frozen-phonon method for state anticrossing situations and its application to zero-point motion effects in diamondoids. *Phys. Rev. B* **2023**, *108*, 125403.

(2) Bürkle, M.; Viljas, J. K.; Hellmuth, T. J.; Scheer, E.; Weigend, F.; Schön, G.; Pauly, F. Influence of vibrations on electron transport through nanoscale contacts. *physica status solidi (b)* **2013**, *250*, 2468–2480.

(3) Han, P.; Bester, G. Heavy strain conditions in colloidal core-shell quantum dots and their consequences on the vibrational properties from ab initio calculations. *Phys. Rev. B* **2015**, *92*, 125438.

(4) Neugebauer, J.; Reiher, M.; Kind, C.; Hess, B. A. Quantum chemical calculation of vibrational spectra of large molecules—Raman and IR spectra for Buckminsterfullerene. *J. Comp. Chem.* **2002**, *23*, 895–910.

(5) Rappoport, D.; Furche, F. Lagrangian approach to molecular vibrational Raman intensities using time-dependent hybrid density functional theory. *J. Chem. Phys.* **2007**, *126*, 201104.

(6) Bhati, M.; Ivanov, S. A.; Senftle, T. P.; Tretiak, S.; Ghosh, D. How structural and vibrational features affect optoelectronic properties of non-stoichiometric quantum dots: computational insights. *Nanoscale* **2023**, *15*, 7176–7185.

(7) Dzhagan, V. M.; Azhniuk, Y. M.; Milekhin, A. G.; Zahn, D. R. T. Vibrational spectroscopy of compound semiconductor nanocrystals. *J. Phys. D: Appl. Phys.* **2018**, *51*, 503001.

(8) Steenbock, T.; Drescher, E.; Dittmann, T.; Bester, G. How Surface Defects Shape the Ex-

citons and Photoluminescence of Ultrasmall CdSe Quantum Dots. *Chem. Mater.* **2024**, *36*, 6504–6514.
