# Supplementary material for: Origin of the High-Frequency Shoulder in the Raman Spectra of CdSe Quantum Dots
Source: J Phys Chem Lett. 2024 Oct 9;15(41):10392–8. doi: 10.1021/acs.jpclett.4c02335 (PMC11492379; doi:10.1021/acs.jpclett.4c02335)
Supplement: Supplementary file 4 — jz4c02335_si_004.pdf [file jz4c02335_si_004.pdf]

jz-2024-02335g.R1

Name: Peer Review Information for "Origin of the High-Frequency Shoulder in the Raman Spectra of CdSe Quantum Dots"

First Round of Reviewer Comments

Reviewer: 1

Comments to the Author

The major advance reported in the paper lies in the fact that it is the first systematic and in-depth theoretical and numerical study of an unusual vibrational (Raman) spectral feature experimentally observed by many authors so far but not given unambiguous assignment.

The results presented by the authors can contribute to buiding a Raman-based a qualitative and quantitative probe of surface defects and surface functionalisation/bonding on small nanocrystals or other nanostructures.

In their work the authors study were profoundly the case of CdSe, and also in the abstract the authors mention that "The origin of the high-frequency shoulder (HFS) observed above the longitudinal optical (LO) peak around 230 cm−1 in the Raman spectra of CdSe quantum dots (QDs) has been a subject of intense debate.". However, a similar spectral feature has been noticed for other II-VI QDs, such as CdS, CdTe, ZnTe [31][https://doi.org/10.1103/PhysRevB.85.085418]. Could the authors comment on that ? In particular whether NCs of all compounds my have a similar origin or each system need to be considered separately.

Reviewer: 2

Comments to the Author

This article points out for the first time the origin of the high-frequency shoulder peak (HFS) in the Raman spectra of CdSe quantum dots by DFT calculations. This is a good article from the point of view of DFT calculations. But I think it lacks the support of experimental proofs. On page 12, third line, I don't see the modeling and calculations about core-shell quantum dots. The structure of the experimentally synthesized quantum dots is complex, and as stated on page 3, line 27 of the article, the quantum dots may be Se-rich on the surface leading to a Raman spectrum that appears

HFS.In addition, I remain skeptical about the accuracy of the calculations in the article. Is the use of the def2-SVP basis set insufficiently accurate?

Author's Response to Peer Review Comments:

Luruper Chaussee 149 · 22761 Hamburg

Senior Editor
The Journal of Physical Chemistry Letters

18. September 2024

Prof. Dr. Gabriel Bester

Fakultät für Mathematik, Informatik und Naturwissenschaften
Fachbereich Chemie
Luruper Chaussee 149
22761 Hamburg

Tel. +49 40 42838-5893 gabriel.bester@uni-hamburg.de http://www.chemie.uni-hamburg.de/pc

Manuscript Revision (ID: jz-2024-02335g): *Origin of the High-Frequency Shoulder in the Raman Spectra of CdSe Quantum Dots* by Surender Kumar, Torben Steenbock, and Gabriel Bester

Dear Editor,

Thank you for your recent communication regarding our manuscript (ID: jz-2024-02335g). We have thoroughly reviewed the two enclosed referee reports. We have carefully considered all the issues in the two referee's reports and revised our manuscript accordingly. Our point-by-point reply to all the referee comments is attached below. We hope that our revised manuscript is now suitable for publication in the Journal of Physical Chemistry Letters.

Seite 2/3

Response to the Report of the Referee

Reviewer 1:

We thank the reviewer for their recommendation to publish our manuscript in JPCL. We address the reviewer's comment as:

Comment: However, a similar spectral feature has been noticed for other II-VI QDs, such as CdS, CdTe, ZnTe [31]. Could the authors comment on that ? In particular whether NCs of all compounds may have a similar origin or each system need to be considered separately.

This is an interesting question that we have tried to address in the new version of the manuscript as follows:

The fact that similar HFSs have been reported for other II-VI QDs [16,31,50] leads to the natural question to whether our defect related HFS could explain these results as well. While we were not able to perform further calculations within this work, the defect vibration can be expected to be present in other II-VIs. Indeed, twofold coordinated S or Te atoms (if present in the structure) could undergo the asymmetric stretching mode described here. In terms of frequency, we would expect to see it on the blue side of the LO peak as well since the defect vibration has similarities with an LO vibration but with a more loosely bound chalcogen atom. However, this remains speculative at the moment.

Reviewer 2:

We thank the reviewer for their valuable comments. We address the reviewer's comment as:

Comment: But I think it lacks the support of experimental proofs. On page 12, third line, I don't see the modeling and calculations about core-shell quantum dots. The structure of the experimentally synthesized quantum dots is complex, and as stated on page 3, line 27 of the article, the quantum dots may be Se-rich on the surface leading to a Raman spectrum that appears HFS.

The results for core/shell structure (labeled as $Cd_{45}Se_{45}$\CdS-PH) are shown in Fig. 4b of the manuscript. For better readability, we have added a short sentence referring to Fig.4 b. Now the referenced line reads as follows: *Additionally, our calculations for the core/shell structure, as presented*

in Fig.4 b, do not exhibit this peak, which is consistent with the experimental findings. Concerning the experimental proof related to the possibility that an amorphous Se atom at the surface may be at the origin of HFS we took the opportunity to test it. We have done calculations on the $Cd_{13}Se_{13}$-MA cluster with the addition of a neutral Se atom (i.e. amorphous Se). We find that the inclusion of a neutral Se atom prompts significant bond reconstruction, forming a Se-Se dimer. The Se-Se dimer defect states appear above the LUMO and not within the band gap. We have compared the Raman spectra for both $Cd_{13}Se_{13}$-MA and $Cd_{13}Se_{14}$MA (with an extra Se atom) and observed that both spectra are very similar and do not show any Raman-active defect vibrations (HFS) around 230 cm^{-1}. We have added a paragraph on p.12 of the preprint version (Sentence starting with *To rule out the possibility [...]*) in the revised manuscript with a discussion and have included the comparison in the supporting information. This explicitly eliminates one more possible origin of the HFS and, we hope, strengthen our conclusion.

In addition, I remain skeptical about the accuracy of the calculations in the article. Is the use of the def2-SVP basis set insufficiently accurate?

In response to this concern, we have performed calculations with a larger basis set def2-TZVP. We observed that the spectra with both basis sets are very similar with only significant change for the lowest frequency ligand wiggling mode (LW in Fig. 3 of the manuscript) which are very soft and unrealistic modes (as described in the manuscript) and of no interest. The relevant higher frequency region is almost identical with both basis sets. We now present the comparison in the revised supporting information and wrote a sentence in the main document about our basis set convergence test..

Thank you. With kind regards

The Authors
